# Flattening filter free VMAT for a stereotactic, single‐dose of 30 Gy to lung lesion in a 15‐min treatment slot

**DOI:** 10.1002/acm2.12829

**Published:** 2020-02-10

**Authors:** Damodar Pokhrel, Lana Sanford, Bhaswanth Dhanireddy, Janelle Molloy, Marcus Randall, Ronald C. McGarry

**Affiliations:** ^1^ Department of Radiation Medicine Medical Physics Graduate Program University of Kentucky Lexington KY USA

**Keywords:** beam‐on time, FFF‐beam, outcomes, single dose, stereotactic lung treatment, VMAT

## Abstract

Cone‐beam CT‐guided single dose of lung stereotactic body radiotherapy (SBRT) treatment with a flattening filter free (FFF) beam and volumetric modulated arc therapy (VMAT) is a safe and highly effective treatment modality for selective small lung lesions. Four‐dimensional (4D) CT‐based treatment plans were generated using advanced AcurosXB algorithm for heterogeneity corrections. 6X‐FFF beam produced highly conformal radiosurgical dose distribution to the target and reduced lung SBRT fraction duration to less than 10 min for a single dose of 30 Gy, significantly improving patient comfort and clinic workflow. Early follow‐up CT imaging results (mean, 8 months) show high local control rates (100%) with no acute lung or rib toxicity. Longer clinical follow‐up in a larger patient cohort managed in this fashion is underway to further validate this treatment approach.

## 1 INTRODUCTION

Due to recent advances in technology, stereotactic body radiotherapy (SBRT) has become standard of care for medically inoperable early stage non‐small‐cell lung cancer (NSCLC) patients.^1^, ^2^, ^3^, ^4^SBRT protocol RTOG‐0915 (Arm 1) allowed a single dose of 34 Gy treatment for early stage I peripheral NSCLC patients when dosimetric criteria were achieved.^5^ Videtic et al^6^ reported long‐term follow‐up data, which revealed no excess late toxicity in either arm (34 Gy in 1 fraction and 48 Gy in 4 fractions), coupled with consistent high rates of local control. The median overall survival of 4 yr for each arm suggests similar efficacy. They concluded that single‐fraction SBRT of 34 Gy remains a suitable treatment option for patients with early stage inoperable lung cancer. In another study, Videtic and colleagues^7^ compared two single‐fraction SBRT dose schemes of 30 Gy and 34 Gy for 80 medically inoperable early stage I NSCLC patients. Both treatment schedules provided equivalent tumor local control and overall survival rates with minimal toxicity. Therefore, a stereotactic, single dose of 30 Gy is an equally effective treatment for the selected NSCLC patients and is gaining popularity.

Treatment delivery developments, including volumetric modulated arc therapy (VMAT) and flattening filter free (FFF) beams have reduced SBRT treatment time significantly and improved patient compliance.^8^, ^9^, ^10^, ^11^ Removal of the flattening filter from the gantry reduces head scatter, out‐of‐field dose, residual electron contamination, and delivers treatments with higher dose rates up to factors of 2.33 for 6X‐FFF and 4 for 10X‐FFF beams compared to the traditional flattened beams.^12^, ^13^, ^14^, ^15^, ^16^ Because of the reduced treatment times, VMAT with FFF beams is particularly appealing for delivering a single large dose of SBRT treatment to lung lesions potentially minimizing intrafraction motion errors as well. Single‐dose SBRT treatment of 30 Gy to a lung lesion is the extreme form of hypofractionation to extracranial lesions we deliver in our clinic. This could potentially result in a great radiobiological effectiveness because of the delivery of a large single dose.^17^ Due to the fast, safe and effective option of the stereotactic treatment of a single large dose of 30 Gy to a lung lesion for selective lung cancer patients, we sought to present our initial clinical experience (implementation) of our FFF‐VMAT lung SBRT technique as well as report early clinical outcomes in patients with medically inoperable early stage NSCLC.

## 2 MATERIALS AND METHODS

### 2.A Patients, treatment planning, and delivery

Thirteen consecutive early stage I–II NSCLC patients underwent single‐dose lung SBRT at our clinic. Tumors were located as follows: seven in the upper and two in the central part of upper lobes of the left lung; and three in the middle and one in the lower lobes of the right lung. Patients were positioned supine with arms above their head using an armrest and abdominal compression and immobilized using Body Pro‐Lok^TM^ platform (CIVCO system, Orange City, IA). Free‐breathing contrast‐free planning CT scans (General Electric Medical Systems, Waukesha, WI) were acquired at 2.5 mm slice thickness followed by a 4D‐CT scan (Varian RPM System, version 1.7). The maximum intensity projection (MIP) from 10 breathing phases was used to derive the internal target volume (ITV). To account for geometric uncertainties, an isotropic planning target volume (PTV) margin of 5 mm was added to the ITV. Mean PTV was 13.0 ± 12.2 cc (range 4.3–41.1 cc) with a corresponding average tumor diameter of 2.7 ± 0.7 cm (range 2.0–4.2 cm). The main organs at risk (OAR) delineated were: bilateral lungs excluding the ITV (normal lung), spinal cord, ribs, heart, big vessels, esophagus, and skin.

All patients were treated with cone beam CT‐guided VMAT with 6X‐FFF (1400 MU/min) beam on Truebeam Linac. VMAT plans were individually designed using multiple noncoplanar partial arcs (3–4 arcs with ± 10–15° couch kicks) chosen to achieve the planning objective for each patient. Patient‐specific collimator rotations and jaw tracking options were used. All dose distributions were computed using the advanced AcurosXB algorithm for heterogeneity corrections with photon optimizer MLC algorithm^18^, ^19^, ^20^ (AcurosXB, version 13.6) implemented in the Eclipse treatment planning system. The calculation grid size was 1.25 mm and dose to medium reporting mode was used. Prescription dose was 30 Gy in 1 fraction; at least 95% of the PTV received the prescription dose and the maximum dose to the PTV was limited to 130% (fall within the ITV) of the prescription dose.

Before delivering each SBRT treatment, a daily quality assurance (QA) check was performed on kilovoltage to megavoltage imaging isocenter coincidence, including IsoCal measurement for precise and accurate target localization. The IsoCal localization accuracy for Truebeam was <0.5 mm. All the quality assurance procedures including patient‐specific QA were in compliance for SBRT treatment delivery.^17^ Patient‐specific VMAT–SBRT QA was performed using an Octavius 4D (PTW, Freiburg, Germany) phantom with an Octavius 1500 detector array insert and the average pass rate was 97.6 ± 2.7% for 3%/2 mm criteria.

**Figure 1:**
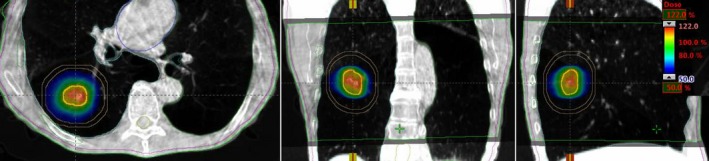
Axial, coronal, and sagittal views of CBCT images (see inset) co‐registered with planning CT images (see back at coronal and sagittal views) used for image‐guided SBRT treatment. In addition to anatomical landmarks, the planned dose cloud was superimposed. CBCT images were acquired in free breathing with abdominal compression, and soft‐tissue three‐dimensional (3D) matching was performed manually. SBRT, stereotactic body radiotherapy.

Patients were initially positioned using external marks and in‐room lasers, followed by pretreatment free‐breathing cone beam CT scan. Every patient setup prior to single‐dose lung SBRT was performed using an in‐house SBRT/IGRT protocol by co‐registering pretreatment cone beam CT with the planning CT scans at the Truebeam Linac (see Fig. 1). Image registration was performed automatically based on region of interest and bony landmarks, followed by manual refining performed by the treating physician to ensure that the tumor was registered with the ITV contoured on the planning CT. The patient position was then corrected for 6 degrees of freedom (DOF) according to the results of soft tissue registration and the treatment was delivered. Those 6‐DOF couch corrections were within the limits of our departmental SBRT protocol guidelines for each patient (translational shifts within ± 2.0 mm and rotational shifts within ± 2.0° in each direction). The patient setup, tumor matching on cone beam CT scan, and treatment delivery were monitored and verified by the treating physician and physicist. Figure 1 shows the planned isodose color wash superimposed with daily CBCT images after the couch corrections were applied.

For comparison of dosimetry and treatment delivery efficiency, all cases were replanned using identical VMAT geometry with traditional flattened 6X‐FF beam with maximum available dose rate of 600 MU/min. The same prescribed dose, planning objectives during plan optimization, and requirement for plan evaluation were used as for 6X‐FFF plans. The major RTOG parameters evaluated for target coverage include:

1. Conformity index (CI): ratio of prescription isodose volume to the PTV. CI less than 1.2 is highly desirable; CI = 1.2–1.5, acceptable with minor deviations.
2. Gradient index (GI): ratio of 50% prescription isodose volume to the PTV. GI has to be smaller than 3–6, depending on the PTV.
3. Maximum dose at any point 2 cm away from the PTV margin in any direction (D_2cm_): D_2cm_ has to be smaller than 50–70%, depending on the PTV size.
4. Percentage of normal lung receiving dose equal to 20 Gy or more (V20Gy): V20Gy should be less than 10% per protocol, V20Gy less than 15% is acceptable with minor deviations. V20Gy is for total lungs minus the ITV.

Furthermore, the gradient distance (GD) was defined as the average distance from 100% prescribed dose to 50% prescribed dose which indicates how sharp the dose falls off. The GD is used to evaluate dose sparing to normal lung volume. The modulation factor (MF) was defined as the ratio of total number of MU to the prescription dose (in cGy). RTOG dose limits for maximum doses to spinal cord < 14.0 Gy, heart < 22.0 Gy, esophagus < 15.4 Gy, maximum dose and dose to 1 cc of ribs, <30.0 Gy and < 22.0 Gy, and maximum dose and 10 cc of skin < 26.0 Gy and < 23.0 Gy, respectively, were used for plan evaluation per single‐fraction lung SBRT protocol (see Arm 1).^5^ Statistical analysis was performed using Microsoft Excel (Microsoft Corp., Redmond, WA) data analysis software. Paired two‐sided Student’s t‐test was used to evaluate parameters for 6X‐FFF vs traditional 6X‐FF plans using a *P*‐value < 0.05 (statistically significant).

## 3 RESULTS

### 3.A Treatment plan characteristics

Steep dose gradients and much faster treatment delivery were achieved with 6X‐FFF VMAT plans. All lung SBRT plans with 6X‐FFF beam were acceptable per RTOG guidelines for the CI and intermediate dose spillage parameters and doses to OAR. The evaluated intermediate dose spillage parameters included GI, D_2cm_, and GD in addition to V20Gy. Although both plans were acceptable per the RTOG standard, the 6X‐FFF plan had advantages of providing radiosurgically tighter intermediate dose spillage (see GI, D_2cm_, GD, significant p‐values in Table 1) compared to traditional 6X‐FF plan. Statistically significant p‐values are shown in bold (see Table 1).

**Table 1:** Plan quality evaluation for clinical 6X‐FFF and traditional flattened 6X‐FF (replanned) plans for all 13 VMAT lung SBRT patients.

Target and V20Gy	Parameters	VMAT (6X‐FF)	VMAT (6X‐FFF)	*P*‐value
PTV	CI	1.09 ± 0.11 (0.98–1.39)	1.07 ± 0.08 (0.98–1.24)	*n.s.*
	GI	5.8 ± 1.3 (3.92–8.11)	5.5 ± 1.1 (3.81–7.23)	***P = 0.001***
	D_2cm_ (%)	50.0 ± 5.5 (38.8–58.8)	48.0 ± 4.7 (37.8–55.1)	***P = 0.001***
	GD (cm)	1.1 ± 0.2 (0.79–1.52)	1.0 ± 0.2 (0.77–1.37)	***P = 0.001***
Healthy lung	V20Gy (%)	0.6 ± 0.4 (0.1–1.6)	0.6 ± 0.4 (0.1–1.5)	***P = 0.004***

Note

The statistical significance at *P* < 0.05 are shown bold. D_2cm_ = maximum dose at any point 2 cm away from the PTV margin in any direction, V20Gy = percentage of normal lung volume receiving 20 Gy or more.

Abbreviations: CI, conformity index, GI, gradient index; GD, gradient distance; n.s., not significant; PTV, Planning target volume; SBRT, stereotactic body radiotherapy; VMAT, volumetric modulated arc therapy.

**Figure 2:**
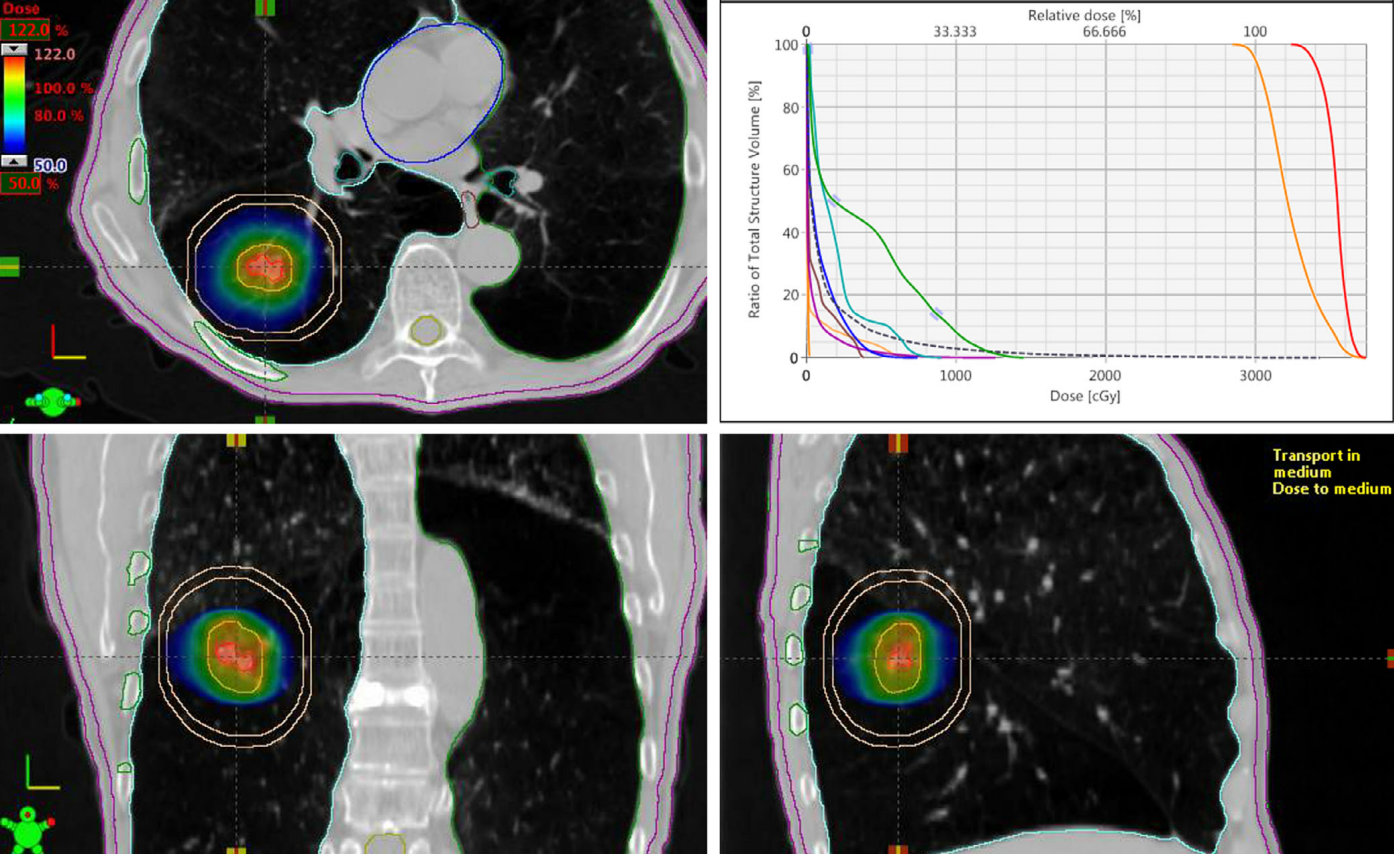
This is a radiosurgical dose distribution in three views (axial‐, coronal‐ and sagittal) and the corresponding DVH for ITV (red), PTV (orange) and OAR for patient #6 treated with noncoplanar VMAT plan. This plan was normalized to deliver PTV D95 full dose, and the blue isodose colorwash (50% isodose spillage) constricted within D_2cm_ around the target volume. PTV was 10.7 cc and located in the right‐middle lobe. The cross‐hair shows the isocenter location and OAR contours of ribs, esophagus, bronchial tree, spinal cord, normal lung, and skin are shown. OAR, organs at risk; PTV, planning target volume; VMAT, volumetric modulated arc therapy.

All other doses to OAR including rib and skin were much lower than RTOG requirement and dosimetrically superior with 6X‐FFF beam compared to traditional 6X‐FF beam (not shown here). For the patient shown in Fig. 2, the PTV was 10.7 cc (2.71 cm diameter) and located in the middle right lung. VMAT plan consisted of four noncoplanar partial arcs (total of 10,090 MU was delivered for a single dose of 30 Gy). Beam on time was 7.21 min with 6X‐FFF beam for the Truebeam Linac. In this case, the VMAT plan gave CI, D_2cm_, GI, GD, and V20 were 1.20, 53.8%, 7.23, 1.37 cm, and 0.6% with 6X‐FFF beam, all parameters within RTOG compliance.

### 3.B Treatment delivery parameters

The dose delivery rates for each control point of the VMAT arcs was recorded 1400 MU/min with 6X‐FFF beam and was verified experimentally during patient‐specific QA delivery at the machine as well as under MLC properties in the Eclipse TPS. Comparison of treatment delivery parameters (total number of monitor units, modulation factor and beam‐on time) are shown in Table 2. It has been observed that for both 6X‐FF and 6X‐FFF plans total number of monitor units and MF were similar. However, the average BOT was improved by a factor of 2.33 when utilizing the 6X‐FFF beam.

**Table 2:** Comparison of average values of treatment delivery parameters: mean ± SD (range) between clinical 6X‐FFF and re‐optimized 6X‐FF plan for all 13 lung SBRT patients.

Delivery parameters	VMAT (6X‐FF)	VMAT (6X‐FFF)	*P*‐value
Monitor units (MU)	9034 ± 2159 (6245–15131)	9040 ± 2045 (6435–14684)	*n.s.*
Beam modulation factor (MF)	3.0 ± 0.72 (2.1–5.04)	3.0 ± 0.68 (2.15–4.89)	*n.s.*
Beam‐on time (min)	15.1 ± 3.6 (10.41–25.22)	6.5 ± 1.5 (4.6–10.5)	***P < 0.001***

Abbreviations: n.s., not significant; SBRT, stereotactic body radiotherapy; SD, standard deviation; VMAT, volumetric modulated arc therapy.

Statistically significant values at *P* < 0.05 are shown in bold.

Estimated mean couch time for a single dose of 30 Gy lung SBRT treatment with 6X‐FFF beam (including CBCT imaging) was 10 min. The average net treatment time (from first beam on until last beam off, including couch kick time) was about 8 min. All patients completed single dose of 30 Gy to the lung lesion.

**Figure 3:**
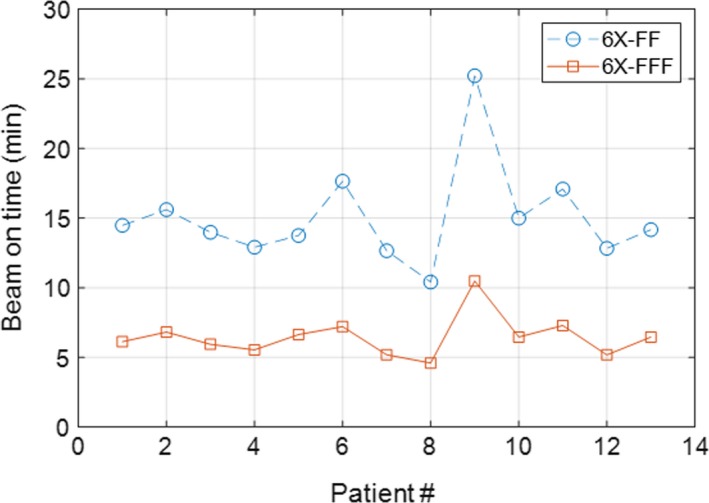
Beam on time for all 13 lung SBRT patients: mean value of beam on time was 6.5 ± 1.5 min with 6X‐FFF compared to 15.1 ± 3.6 min with 6X‐FF VMAT plans, with an absolute reduction of beam on time by 8.6 min, on average (maximum up to 14.73 min), for a single dose of 30 Gy lung SBRT treatment. SBRT, stereotactic body radiotherapy; VMAT, volumetric modulated arc therapy.

The variation in BOT for 6X‐FF and 6X‐FFF plans on a per patient basis is also shown in Fig. 3. For the single dose of 30 Gy, range of BOT for 6X‐FFF was 4.6–10.5 min, much shorter than 6X‐FF beam of 10.41–25.22 min and hence significantly affecting the patient’s treatment time (see *P*‐value in Table 2).

### 3.C Clinical follow‐up outcomes

All 13 lung SBRT patients are alive at the time of this review (Table 3). Median follow‐up time was 8 ± 4 months (range, 3–15 months). All patients achieved complete response to treatment with no reported treatment related lung or rib toxicity. Patient follow‐up included physical exam followed by CT scan every 3 months for the first year and then as clinically indicated. Table 3 shows the tumor local control rates and toxicity profiles. Of the 13 patients, only two patients showed progression of disease that had occurred as distant metastases: one with an endobronchial recurrence and another one with a right upper lobe lesion; however, both treated lesions disappeared (see patient #1 and patient #12, Table 3).

**Table 3:** Treatment outcomes for 13 lung SBRT patients treated with 6X FFF‐VMAT for a single dose of 30 Gy.

Pt. no.	Tumor location	Local control (months)	Recurrence	Lung toxicity	Rib toxicity
			Local/distant Time to progress	Acute/grade Late/grade	Acute/grade Late/grade
1	Central part of upper, left lung	6	Endobronchial distant, 6 months	Grade 1	None
2	Central part of upper, left lung	11	None	Grade 1	None
3	Upper, left lung	8	None	Grade 1	None
4	Lower, right lung	9	None	Grade 1	None
5	Upper, left lung	3	None	Grade 1	None
6	Middle, right lung	9	None	Grade 1	None
7	Upper, left lung	3	None	None	None
8	Middle, right lung	15	None	Grade 1	None
9	Upper, left lung	12	None	Grade 1	None
10	Upper, left lung	3	None	None	None
11	Upper, left lung	6	None	Grade 1	None
12	Middle, right lung	6	Right upper lobe distant, 6 months	Grade 1	None
13	Upper, left lung	6	None	Grade 1	None

Abbreviations: SBRT, stereotactic body radiotherapy; VMAT, volumetric modulated arc therapy.

The Kaplan–Meier estimated 1‐year actuarial tumor local‐control rate was 100%. During the follow‐up period, no patient had pulmonary adverse events and no patient developed grade 2 or higher pneumonitis or chest wall pain/rib fracture. However, 11 patients developed radiographic changes of evolving lung fibrosis (grade 1 pneumonitis) that were asymptomatic in nature.

## 4 DISCUSSION

Very fast and RTOG‐0915‐compliant treatment planning and delivery using 6X‐FFF beam VMAT plans for a single dose of 30 Gy lung SBRT was presented. Furthermore, 6X‐FFF VMAT provided a dosimetrically superior treatment plan with tighter intermediate dose spillage, lower dose to OAR, and much faster treatment delivery. Much tighter radiosurgical dose distributions with 6X‐FFF beam were due to the unique beam profile, softer energy spectrum, smaller out‐of‐field scatter and leakage characteristics compared to traditional 6X‐FF beam as discussed above. The main advantages of the 6X‐FFF VMAT plan was significant reduction of BOT. Compared to traditional flattened 6X‐FF beam, the total number of MU did not change significantly while using 6X‐FFF beams, suggesting that both plans had similar plan complexity and hence provide similar beam modulation. However, due to the faster dose rate, the average BOT for 6X‐FFF VMAT plan was 6.5 ± 1.5 min that was much shorter than 6X‐FF VMAT plan (15.1 ± 3.6 min) and hence significantly affecting the overall treatment time.

The use of single‐fraction lung SBRT treatments has been previously studied.^5^, ^6^, ^7^, ^21^, ^22^, ^23^, ^24^, ^25^, ^26^, ^27^ For instance, Siva et al^24^ reported comparable local control rates, overall survival and toxicity profiles between single dose of 26 Gy for peripheral tumors and 18 Gy for centrally located tumors compared to 48 Gy in 4 fractions for peripheral tumors and 50 Gy in 5 fractions for the central tumors for patients with FDG‐PET staged pulmonary oligometastases. Recently, the results of a randomized phase‐II trial of 30 Gy in 1 fraction versus 60 Gy in 3 fractions for medically inoperable early stage lung cancer patients showed similar toxicity and effectiveness between the two arms.^26^ While comparing single fraction to 3 fractions schemes it was reported that 8 (16%) and 6 (12%) patients on each arm, respectively, developed grade 3 lung toxicity. However, their lung SBRT treatment plans did not use heterogeneity corrections and treatment time was not reported. The same group of investigators also recently reported long‐term (8‐year) follow‐up results comparing the same single dose of 30 Gy vs 3 fraction dose of total 60 Gy schemes for 159 lung SBRT patients and found similar outcomes.^27^ Our noncoplanar VMAT lung SBRT planning strategy used advanced Acuros‐based dose calculation for heterogeneity corrections and further optimized treatment plans for higher tumor dose (120%–130% hotspot in the middle of the ITV) and steep dose falloff outside the PTV, allowing much lower dose to normal lung, rib or skin and no reported acute toxicity.

The interplay effect with change in breathing motion with the MLC modulation and gantry rotation in the delivery of FFF‐VMAT plan can be a concern for single‐dose lung SBRT treatment. However, it has been demonstrated that the interplay effect causes negligible dose blurring when using two or more VMAT arcs for FFF beam.^28^ Our treatment planning strategy utilized 3–4 noncoplanar VMAT arcs that could reduce the dose blurring. Furthermore, the change in respiratory patterns between the planning CT simulation and the time of treatment has been studied previously.^29^, ^30^ Although it has been reported that there were only small changes (within ± 3 mm) due to intrafractional and interfractional motion in lung SBRT treatments, the mean patient set up time from tumor localization to the end of treatment cone beam CT scan was about 40 min.^30^ It was recommended that an isotropic 5 mm PTV margin around the ITV was sufficient to address these potential motion errors. In this study, while using 4D treatment planning‐based ITV concept (treating patient on 100% duty cycle), our average beam on time of 6.5 min for a single dose of 30 Gy lung SBRT treatment decreases the variation of intrafraction motion error due to coughing or pain, making geographic miss less likely and improving the patient stability and clinic workflow.

With frequent use of FFF beam for stereotactic treatment, the instantaneous dose rate has increased by approximately a factor of 2.33 for our lung SBRT cases treated with 6X‐FFF beam. It could increase up to by a factor of 4 with 10X‐FFF beam. It is not currently clear to whether these higher dose rates could impact radiobiological effectiveness for tumor control and normal tissues toxicity.^31^, ^32^, ^33^ Further investigation and clinical trials with close follow‐up are needed to assess any radiobiological consequences of flattened vs unflattened beams. However, in our initial clinical experience, single‐dose lung SBRT is shown to be a fast and safe treatment, improving patient’s compliance and clinic flow, potentially lowering treatment cost and, at this point, appears equally as effective as other hypofractionated lung SBRT techniques.

## 5 CONCLUSION

6X‐FFF noncoplanar VMAT plans for a single dose of 30 Gy lung SBRT provided fast and effective treatment with tighter radiosurgical dose distributions to the target, lower intermediate dose‐spillage and lower dose to OAR compared to traditional 6X‐FF beam while significantly reducing BOT by a factor of 2.33 (on average, 6.5 min with 6X‐FFF vs 15.1 min with 6X‐FF beam). With 6X‐FFF VMAT, treatment could be delivered in a 15‐min slot at the Truebeam Linac. Early clinical follow‐up results (mean, 8 months) are promising with 100% local tumor control rate and no reported treatment related lung or rib toxicity. Long‐term clinical follow‐up is underway to access late toxicity and definitive tumor control outcome of this treatment approach.

## CONFLICT OF INTEREST

None.
